# Effect of voice therapy in sulcus vocalis: A single case study

**DOI:** 10.4102/sajcd.v63i1.146

**Published:** 2016-11-30

**Authors:** R. Rajasudhakar

**Affiliations:** 1 Department of Speech-Language Sciences, All India Institute of Speech and Hearing (AIISH), India

## Abstract

**Background:**

Sulcus vocalis is a structural deformity of the vocal ligament. It is the focal invagination of the epithelium deeply attaching to the vocal ligament. There is a dearth of literature on the outcome of voice therapy in sulcus vocalis condition.

**Objective:**

The primary objective of this study was to document voice characteristics of sulcus vocalis and the secondary objective was to establish the efficacy of voice therapy in a patient with sulcus vocalis.

**Method:**

A trial of voice therapy was given to the client who was diagnosed as having sulcus vocalis. Boon’s facilitation techniques were used in voice therapy along with other techniques such as breath holding and push and pull approach prior to surgery. Acoustic, aerodynamic, perceptual, quantitative measures of voice quality and self-rating measurements were performed before and after voice therapy.

**Results:**

Improvement was noticed in 10/10 acoustic, 4/4 aerodynamic, perceptual, dysphonia severity index and voice handicap index scores, which hinted that voice therapy can be an option critically for clients with sulcus vocalis in the initial stage.

**Conclusion:**

Voice therapy showed promising improvement in the study and it must be recommended as the initial treatment option before any surgical management.

## Introduction

Speech is the most frequent and significant way in which humans use language to communicate. Speech contains fluency, articulation and voice as its components. The systems included in the production of voice are phonatory, respiratory and resonatory systems. Pitch, loudness and quality are the three parameters of voice. Any deviance in these parameters from normality results in voice disorders. Abnormal voice is any voice that calls attention to itself, does not meet the occupational or social needs of the speaker, or is inappropriate to the age, gender or situation (Aronson & Bless, 2010). Voice disorders are mainly classified as organic and functional voice disorders.

Organic voice disorders could lead to structural changes in the voice production system. Sulcus vocalis is one of the uncommon clinical conditions caused by structural abnormalities in the vocal folds. It is the focal invagination of the epithelium deeply attaching to the vocal ligament (Bouchayer & Cornut, 2000). The lack of tissue causes a divot in the vocal fold which gives the disorder its medical name ‘sulcus’, which means ‘cleft’ or ‘furrow’ (in Latin). Sulcus vocalis is a groove mainly along the edge of superficial lamina propria. In severe cases, the groove can extend up to the intermediate and the deep layer also. There are three types of sulcus vocalis: physiologic sulcus, sulcus vergeture and sulcus vocalis proper (Ford, Inagi, Bless, Khidr & Gilchrist, 1996). Physiologic sulcus (type 1) is a longitudinal depression that extends along the superficial layer of lamina propria without actually moving the vocal ligament. In physiologic sulcus, there is preserved vibratory activity and anatomic layer of lamina propria as reported. Sulcus vergeture (type 2) is a more extensive longitudinal indentation that does not extend into the vocal ligament, involving loss of superficial, intermittent and deep layer of lamina propria. Sulcus vocalis proper (type 3) is a focal pit which extends beyond the vocal ligament into the thyro-arytenoid muscle (Bouchayer & Cornut, 2000).

### Incidence and prevalence

About 15 patients (1.07%) were diagnosed with sulcus vocalis among 1400 patients with voice disorders in Denmark (Greison 1984). The prevalence of sulcus vocalis was estimated in excised vocal folds among laryngeal cancer patients in the USA (Nakayama, Ford, Brandenburg & Bless, 1994). These authors observed 58 cancer patients and reported 28 (48.3%) had sulcus deformities. Bilateral sulcus was found in 7 of 28 (25%) cancer patients and unilateral sulcus was found in 21 of 28 (75%) cancer patients. The authors even compared the presence of sulcus in non-diseased control patients with cancer patients and concluded that sulci were common in cancer group (Nakayama *et al*., 1994).

### Aetiology

The cause of this disorder is not widely studied and is poorly understood. The three causes for sulcus vocalis that are reported in the literature are congenital, acquired and unknown causes (Postma, Blalock & Koufman, 1998). The congenital cause includes faulty development of the fourth and sixth branchial arches and rupture of the epidermoid cyst and vocal fold scars are characterised by replacement of the normal micro-architecture by disorganised collagen. Acquired causes are associated with vocal abuse, laryngoesophageal reflux, trauma and infections.

### Signs and symptoms

All the measures of voice such as perceptual, aerodynamic, physiological and acoustics of voice are affected in patients with sulcus vocalis. The clinical features of the disorder include reduced phonation time (*aerodynamic*), altered fundamental frequency (*acoustical*), dysphonia, breathiness, harshness, hoarseness of voice (*perceptual*), vocal fatigue, incomplete glottal closure, interrupted mucosal wave transmission and ‘spindle-shaped’ glottis (*physiology*) (Boon, McFarlane, Von Berly & Zraick, 2010; Hirano, Yoshida, Tanaka & Hibi, 1990).

### Treatment

There are two lines of management: medical (surgical) and non-medical (behavioural modification, counselling and voice therapy) management. Surgical management includes vocal fold medialisation which includes intrafold injection and medialisation surgery. Intrafold injection includes transoral injection with indirect laryngeal mirror, transoral injection with direct laryngoscopy and transcutaneous injection. The medialisation surgery includes surgical augmentation, medial shift of thyroid cartilage and rotation of arytenoid cartilage (Calton & Casper, 1990). However, studies report that the impacts of surgeries in sulcus are unpredictable and the goal is usually to reduce the glottic leakage. It may also be possible that the post-operative voice would be even worse than the preoperative voice (Giovanni, Chanterect & Lagier, 2007).

Voice therapy includes implementing hygienic, symptomatic, psychogenic, physiologic and eclectic approaches. Voice therapy has limited success in clients with sulcus vocalis (Rubin & Yanagisawa, 2006). In general, voice therapy in sulcus vocalis focuses on reducing strain on vocal folds, reducing compensatory voice changes and optimising the voice production subsystems, thereby increasing the vocal efficiency. However, there is a need to systematically document the voice profile of clients with sulcus vocalis in order to understand their voice characteristics and determine the efficacy of voice therapy for such individuals.

## Aim of the study

This study aimed at determining the effect of voice therapy in a client with sulcus vocalis.

### Client history

A 23-year-old adult male ‘C’ who completed a Bachelor of Engineering (BE) and was working in a private firm as an automobile supervisor reported to the Department of Clinical Services at All India Institute of Speech and Hearing (AIISH), Mysore, with the complaint of his voice being wrongly perceived as female voice. No pubertal change of voice was reported. Difficulty in varying the pitches while speaking was reported. He reported that the amount of voice usage at work settings was about 6 h. Also, he complained of vocal tiredness after excessive use of voice. In addition, he reported that the level of noise in the automobile workshop was moderate. No other associated problems were reported. The aim and objective of the study was explained to the client and a written consent was obtained before initiation of the study.

### Voice evaluation

A detailed voice assessment was carried out by a team of professionals including an otorhinolaryngologist, a speech-language pathologist and a phonosurgeon.

**FIGURE 1:**
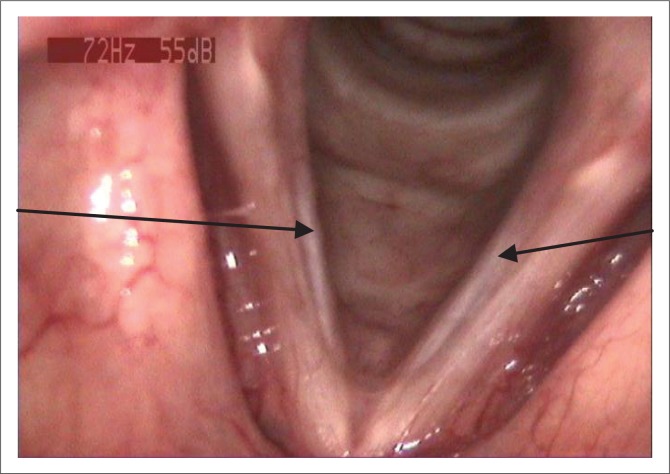
Bilateral sulcus vocalis of vocal folds (pointed by the arrow marks).

*Otorhinolaryngologist’s and phonosurgeon’s evaluation:* Fibre-optic flexible laryngoscopy (FFL) was used to view the vocal tract and glottis. The client was diagnosed to have bilateral sulcus vocalis (Figure 1).

#### Evaluation by speech-language pathologist

A detailed evaluation by a speech-language pathologist consisting of acoustic, aerodynamic, perceptual and quantitative measures of voice quality such as dysphonia severity index (DSI) (Wuyts *et al*., 2000) and self-rating measures using voice handicap index (VHI) was carried out. Acoustic voice evaluation was carried out by using Vaghmi software (VSS, Bangalore) in a sound-treated room where ‘Shure’ SM 48 model microphone was used. Aerodynamic measures were performed using a spirometer (Helios, 701 model from RMS, Chandigargh). lingWAVES (WAVOSYS, Germany) software was employed to measure DSI scores. Perceptual evaluation of voice was carried out by using GRBAS scale (Hirano 1981). Two experienced speech-language pathologists, who had 10 years of experience in assessing and treating clients with voice disorders, assessed perceptually the phonation as well as spontaneous speech sample of client ‘C’. Pre- and post-therapy phonation and/or speech samples were randomised for perceptual evaluation, where the judges were blindfolded for the purpose. They were asked to rate samples on a four-point rating scale using the GRBAS scale. The psychosocial impact of dysphonia because of sulcus vocalis was assessed using voice handicap index (VHI) (Jacobson *et al*., 1997) questionnaire (which consisted of 30 questions). It is a self-rating scale that highlights the perceived severity of voice problem by the client. The voice evaluation was performed two consecutive days before the initiation of voice therapy and the results were averaged. The voice evaluation results are presented in Table 1. These measures were obtained before the initiation of voice therapy programme (pre-therapy).

**TABLE 1:** Comparison of pre-therapy and post-therapy voice parameters.

SI. Number	Parameters	Pre-therapy	Post-therapy
1	Average F0	181.6 Hz	138.3 Hz
2	SD of F0	8.79 Hz	6.93 Hz
3	Jitter	0.23%	0.21%
4	Shimmer	0.08%	0.03%
5	Extent of fluctuation in F0	28.39	12.5
6	Speed of fluctuation in F0	9	4
7	Extent of fluctuation in I0	5.9	3.3
8	Speed of fluctuation I0	10	6.8
9	NNE	-10.38	-5.93
10	Number of harmonics	2	10
11	S/Z ratio	1.3	1.1
12	FVC	1760 mL	2000 mL
13	MAFR	300 mL/s	266 mL/s
14	Average MPD	6.3 s	13 s
15	DSI	-2.6	4
16	VHI	27	17

SI, Serial number; F0, Fundamental frequency; SD, standard deviation; I0, Intensity; NNE, normalised noise energy; S/Z, Ratio of sustained phonation of phoneme /s/ and sustained phonation of phoneme /z/; FVC, forced vital capacity; MAFR, mean air flow rate; MPD, maximum phonation duration; DSI, dysphonia severity index; VHI, voice handicap index.

### Voice therapy programme

The secondary focus of this study was to determine the effect of non-medical treatment (i.e. voice therapy) for this pathological condition. Boone’s facilitation techniques (Boon *et al*., 2010) including elimination of abuses, relaxation, establishing a new pitch, forward focus and auditory feedback were employed in the voice therapy programme. The aim of the voice therapy plan was: (1) to increase the vocal efficiency by improving the coordination between respiratory and phonatory systems, (2) to improve the quality of voice by forward focus and resonance voice therapy and (3) to eliminate the vocal abusive behaviours through a customised vocal hygiene programme for the client.

The client attended voice therapy for 15 sessions, where each session lasted for about 45 min. Furthermore, the laryngeal adduction exercises including breath holding technique and pushing and pulling technique were practiced in the therapy sessions. The post-therapy measures on all the domains (acoustic, aerodynamic, perceptual, self-rating and objective voice quality measures) were obtained after 15 sessions of voice therapy, and pre- and post-therapy measures on all the domains were compared (Table 1).

## Ethical considerations

Ethical clearance was obtained from the Ethics Committee for Bio-Behavioural Research Projects involving human subjects, at All India Institute of Speech and Hearing (AIISH). Reference number: ECC-Res.art/02/2016-17.

## Results and discussion

The results are discussed in the following two sections: (1) voice characteristics in client ‘C’ and (2) effect of voice therapy. Table 1 shows the comparison of pre- and post-therapy voice measures.

### Voice characteristics in client ‘C’

In the pre-therapy assessment, from Table 1, the average fundamental frequency of the client was 186.6 Hz, which was measured for phonation sample. The obtained frequency value is higher than the male frequency range (Eguchi & Hirsh, 1969). This higher fundamental frequency (F0) led to perceive or recognise the client as female by listeners. The standard deviation (SD) of F0 was 8.79 Hz. The jitter and shimmer values were 0.23% and 0.08%, respectively. The extent and speed of fluctuation in F0 and intensity (I0) was higher in the client when compared to the normative value (ideally it must be zero). The normalised noise energy (NNE) was -10.38 and the number of harmonics was just two in the pre-therapy voice sample.

The forced vital capacity (FVC) and mean air flow rate (MAFR) were measured during pre-therapy assessment and the values were 1760 mL and 300 mL/s, respectively. The FVC of the client was less when compared to the normative value of Indian adult male values (2500 mL and above). Also, maximum phonation duration (MPD) in the client was about 6 s. This indicates poor respiratory support for voice production in the present client of interest. On the other hand, the client had higher MAFR value compared to normal value (80 mL/s – 180 mL/s), which indicates glottal incompetence, that is, poor contact or closure of the vocal folds during phonation (Baken 1987).

The results of the perceptual voice evaluation revealed that judge 1 and judge 2 rated the overall grade of dysphonia as moderate (2) and mild (1), respectively. Breathiness was rated as severe (3) and moderate (2) by judges 1 and 2, respectively. The other parameters such as roughness, asthenia and strain were rated similarly by both the judges at the pre-therapy condition. Table 2 shows the perceptual ratings of both judges at pre- and post-therapy conditions. The measured DSI score (objective voice quality measure) was -2.6 during pre-therapy assessment, indicating a poor voice quality.

**TABLE 2:** GRBAS results before and after voice therapy.

GRBAS scale	Pre-therapy		Post-therapy	
	Judge 1	Judge 2	Judge 1	Judge 2
Grade	2	1	0	0
Roughness	1	1	0	0
Breathiness	3	2	1	0
Asthenia	1	1	0	0
Strain	2	2	0	0

The negative impact of the voice disorder or difficulty was assessed by using VHI-30 questionnaire. The VHI questionnaire is a self-rating questionnaire consisting of 30 questions that assesses the functional, physical and emotional aspects of the client because of voice difficulty.

### Effect of voice therapy

The client attended 15 sessions of voice therapy, and on the 16th session, post-therapy voice assessment was performed by considering the average performance of three trials. The comparison of pre- and post-therapy voice assessments on acoustic, aerodynamic, perceptual, objective voice quality and self-rating measures was carried out (see Table 1).

The average F0 was reduced from 181.6 Hz to 138.3 Hz after voice therapy which now falls within the normal F0 range of adult males (Eguchi & Hirsh, 1969). Also, there was a reduction noticed in the SD of F0. The reduction in SD F0 value indicates reduced variability of pitch. The jitter and shimmer values (perturbation measures) reduced after voice therapy in the client. This indicates more stable voice with less fluctuation in pitch and loudness parameters. Furthermore, the extent and speed of fluctuations in both F0 and I0 was reached relatively within normal limits after voice therapy. The NNE value has improved after voice therapy and also the number of harmonics has increased from 2 to 10 in the post-therapy. In general, it is known that there is a correlation between voice quality and the number of harmonics (Qi & Hillman, 1997). Increased number of harmonics after therapy in client ‘C’ can be attributed to improved and better voice quality.

Forced vital capacity and MPD measurement increased after voice therapy and it needs to be worked upon further to reach the normative range. S/Z ratio and MAFR reduced prominently after therapy. Reduction in MAFR indicated that the airflow between the vocal folds (glottis) during phonation is reduced and it could be because of improvement in the adduction of vocal folds during phonation. Perceptually, the post-therapy sample has no strain and roughness. Judge 2 rated the post-therapy sample as normal (indicated ‘0’ on all the five parameters) in comparison to judge 1 who rated the sample as mild breathy. A prominent reduction was noticed on overall grade, roughness, asthenia and strain by both the judges, which indicated that the client produces voice (both phonation and speech task) with utmost ease without any difficulty or straining himself within the post-test situation.

The DSI value predominantly improved after voice therapy. That is, it improved from -2.6 to 4. The improvement in DSI value could be because of increased MPD value and greater control on pitch and loudness of voice.

The VHI values were also reduced from 27 to 17 after therapy. The decreased VHI values can be attributed to the improvement in functional, physiological and emotional aspects of the client, which is triggered by voice therapy.

Outcome studies after behavioural intervention are very few in the case of sulcus vocalis. In this regard, there is a study wherein the efficacy of a trial voice therapy (10 sessions of 40 min duration for 15 days) in a 31-year-old male client with sulcus vocalis was examined (Ranjini, Ramya, Namboothri & Gopikishore, 2011). In the above study, the authors found positive changes in the vocal behaviours after voice therapy on objective, perceptual and self-perceptual domains. The authors concluded the behavioural voice management such as respiratory–phonatory control exercises, laryngeal manual therapy and forward focus as effective options for treating individuals with sulcus vocalis. The results of the present study are in consonance with the above research findings. However, sulcus vocalis condition is challenging to treat because of the lesions involving the vocal fold layers. Eventually, in general, the physiologic sulcus (type 1) condition responds to voice therapy unlike types 2 and 3. The improvement shown by client ‘C’ leads us to opine that the sulcus vocalis might probably be of type 1 rather than of other types.

## Conclusion

This study was aimed at determining the effect of voice therapy on a client with sulcus vocalis. Also, the study documented and/or profiled the voice characteristics using some of the acoustic, aerodynamic, perceptual, objective voice quality measure (DSI) and self-rating scale (VHI) parameters. The outcome of the voice therapy was empirically documented in the study by comparing the pre-therapy and post-therapy voice parameters.

The study found prominent positive changes in the voice parameters after voice therapy, such as reduction in F0, reduction in perturbation measures (jitter and shimmer), increased number of harmonics, increased MPD, vital capacity and reduced MAFR. The VHI scores were also reduced after therapy. Perceptually, overall grade and strain in voice reduced during post-therapy condition. The objective voice quality measure (DSI) shifted from negative value towards positive value. These changes can be attributed to the influence of non-medical management, that is, voice therapy. Boon’s facilitation techniques, improving the coordination between respiratory and phonatory systems and educating on vocal hygiene, were proved to be beneficial for client ‘C’ with sulcus vocalis. The other contributing factor could be the motivation of the client. The client in this study was greatly motivated and attended all the voice therapy sessions without fail. It is substantial to document systematically the multidomain voice measures for evidence-based clinical practice in voice disorders. Limitations of the study are the following: it is a single-subject study; therefore, one must be cautious in generalising the results and post-therapy FFL could not be done for better comparison because of implicit reasons. The voice therapy employed in the study improved client C’s physiology of vocal apparatus and not the anatomy of vocal folds which would have been commented if FFL was done during post-therapy.

However, this study documented the immediate effect of voice therapy on multiparametric approach in voice assessment. The long-term effect of the same can be studied in future. More studies are warranted in voice therapy outcome measures with more clients with sulcus vocalis. To conclude from this study, voice therapy can be considered as the initial treatment option before any surgical management, and if it fails, the latter can be recommended consequently for clients with sulcus vocalis.
